# Surgery

**Published:** 1892-06

**Authors:** W. H. Harsant


### SURGERY.

Of late years few subjects have been more investigated and experimented with by surgeons than the various means of uniting two divided portions of intestine. Bishop [2] has collected no less than thirty-three distinct methods of doing this, but those in present use may be divided into three: first, the end-to-end method, in which the edges of the cut bowel are somewhat turned in, so as to bring two peritoneal surfaces into contact; second, the method of intestinal anastomosis, by which the walls of two portions of bowel are approximated at some convenient point, and then sutured together with Senn's decalcified bone plates; and third, the method of invagination, founded upon those cases which

[1] *Rev. de Chir.*, Jan. 1, 1891. [2] *Med. Chron.*, Sept., 1885.

have been occasionally observed where recovery has ensued after an intussusception, owing to the intussuscepted bowel having become firmly united to the portion of bowel enclosing it.

It is not yet by any means certain which is the best of these methods, but the following papers are well worthy of consideration.

Under the title of "A New Method of Intestinal Surgery," Dr. H. Widenham Maunsell, lecturer on surgery in Otago University, New Zealand,[1] describes his method of uniting the divided ends of intestine after resection of a portion. He first clamps the bowel to be excised by placing a small flat sponge across the intestine, about four or six inches from the part to be excised, and then transfixes the sponge and the mesentery close to the gut with a strong safety-pin. The pin is then passed again through the sponge on the other side of the gut and clamped. The sponge should be sufficiently large to compress the intestine against the pin, so as to effectually prevent extravasation of the contents. The advantages of this method are its extreme simplicity, its easy applicability, its innoxiousness, and its efficiency. The pressure can be regulated by the size of the sponge. When the entire circumference of the gut is involved in the wound, the bowel must be invaginated so as to bring the encircling peritoneal surfaces into accurate juxtaposition all around, and then sutured in that position. The two ends of the bowel are first brought together with two temporary sutures passed through all the coats of the intestine. The long ends of these sutures are left intact. One is placed at the mesenteric attachment of the gut and the other exactly opposite. These temporary sutures secure the complete peritoneal covering of the mesenteric attachments of both segments of the gut, help to maintain the proper relative position and accurate co-adaptation of the two cut ends, and facilitate their subsequent invagination through the opening made in the larger segment of gut. An opening is then made in the larger segment of the gut, through which the invaginated ends of the divided bowel are dragged by the long ends of the temporary sutures. The edges of this opening in the bowel, which should begin about an inch from its transverse section, should be afterwards well turned in and brought together with a continuous suture passed through the peritoneal and muscular coats only. The description of the method can only be properly understood by a careful study of the series of excellent diagrams which

[1] *Amer. Journ. Med. Sci.*, March, 1892.

accompany the article. The author concludes by some remarks on the simplicity of the operation and the universality of its application to all parts of the intestinal tract. He says it is the only operation as yet devised where rapid circular enterorrhaphy (the sutures passing through all the coats of the bowel, leaving the knots on the inside) can be accomplished. The most important element of success, next to the efficiency of the operation, is its speedy performance. After the bowel to be resected has been isolated with the sponge clamps, circular enterorrhaphy can be accomplished in ten minutes, which is less than half the time required for lateral apposition (intestinal anastomosis) with decalcified bone plates. Horsehair or fine silk-worm gut is recommended as the best material for the sutures.

Mr. F. B. Jessett[1] gives a description of a method of performing enterorrhaphy which he has been led to adopt after numerous experiments on the cadaver. He uses two tubes of decalcified bone, one of which he calls the male tube, and the other the female. The male tube consists of two parts: the cylindrical portion, which is fastened into the proximal end of the intestine; and the spur, which slides into the female tube. The latter is of a conical shape, and measures one inch and a quarter in length; it is inserted into the distal portion of the intestine. Both tubes are fixed to the respective portions of bowel by chromic gut, passed through holes made for the purpose in the bone tubes. The two divided ends of intestine are now approximated by passing the spur of the male tube into the female, and they are tied in this position by the threads attached to the tubes. The surgeon next steadies the proximal end of the intestine over the tube between his left thumb and finger, while with the thumb and finger of the right hand he gently slips the distal part of the intestine over the proximal for a quarter to half an inch. He then, with four quilt chromic gut sutures, fastens it in place, being careful to insert one suture on each side of the mesenteric border.

It is of importance that the tubes should slip quite easily into the intestine, as if they fit at all tightly there is much more difficulty in invaginating the one portion of intestine over the other; moreover, there is the risk of gangrene, as the tubes may swell somewhat after their introduction. Six experiments upon dogs were performed by Mr. Jessett, with the assistance of Dr. Morotti of Milan, at the laboratory of the Royal Colleges of Physicians and Surgeons. The first

[1] *Brit. Med. Journ.*, April 2, 1892.

experiment failed, on account of the tubes being too large and causing compression; but the others were all successful.

Mr. Frank T. Paul[1] points out that the operation of enterorrhaphy, as described by him[2], embraces all the salient points of Mr. Jessett's operation. The modifications used by the latter are: (1) the use of two bone tubes instead of one, these being also longer and heavier than those used by Mr. Paul; (2) the greater number of threads used. Mr. Paul considers these modifications to be no improvements.

The same surgeon reported to the Clinical Society of London, at its meeting on May 13th last, a successful case of his own operation.[3]

The patient was a woman, aged 37, in whom a strangulated hernia had gone unrelieved for at least six days. The bowel was sloughing, and had to be excised. It was reunited by the method of invagination referred to above. The patient was fed freely with fluid nourishment from the first. Solid food was commenced, in the form of bread and milk, on the sixth day, and meat on the fourteenth day. On the eighth day the bowels were well moved by glycerine enema, and subsequently at intervals of a few days. The pulse and temperature remained practically normal, and there were no symptoms worth recording. In three weeks she was allowed up, and at the end of a month left the hospital quite convalescent.

In connection with the same subject, Mr. Arthur E. Barker[4] records a second successful case of gastro-enterostomy. The operation was done for carcinoma of the pylorus, which had given rise to great emaciation, and threatened death by starvation. The stomach was first washed out with large quantities of hot water, followed by a solution of salicylate of soda, ten grains to an ounce. A 4-inch incision was made in the middle line, above the umbilicus, and all bleeding points having been checked, the peritoneum was opened. The growth was immediately seen beneath the wound, seated in the anterior wall of the stomach, its centre being about one and a half inches from the pylorus.

The anterior wall of the stomach having been caught up and held by an assistant, was then opened by an incision about one and a half inches long, and a similar incision made in the jejunum about twelve or fifteen inches from its origin. Senn's plates were then inserted, and brought together by silk sutures in the usual way. The walls of the viscera were

[1] *Brit. Med. Journ.*, April 9, 1892. [2] *Lancet*, vol. i., 1891, p. 1196.
[3] *Lancet*, May 21, 1892. [4] *Brit. Med. Journ.*, Jan. 9, 1892.

afterwards sutured round the plates by continuous sutures of fine silk. The junction now seemed secure, and after careful toilet of the surrounding parts, the wound was closed in the usual manner by hard twist silk.

The operation lasted 48½ minutes, and the patient recovered well. No food was administered for 21 hours, and then a teaspoonful of peptones was given by the mouth every two hours, and a peptonised beef and milk suppository every six hours, alternately with an injection of plain hot water every six hours, only five ounces at a time.

The dressings were removed in ten days, when the wound had healed. The patient lived six weeks after the operation, and then died of acute pneumonia at a convalescent home. At the *post mortem* examination it was found that good union had taken place between the stomach and jejunum, while a circular aperture, five-eighths of an inch in diameter, existed between them. The mucous membrane of the stomach and bowel were continuous over the margin of the aperture, and no line of demarcation could be detected. Nothing could be seen anywhere of Senn's plates. There was no tendency to kinking of the jejunum at the attached point, and no obstruction to the flow of contents at the point attached to the stomach.

W. H. HARSANT.

